# Patient-reported outcomes in large vessel vasculitis: insights from a retrospective analysis of disease activity and associated factors

**DOI:** 10.1186/s41687-023-00681-w

**Published:** 2024-01-08

**Authors:** A. Kernder, M. Rohde, H. Acar, C. Düsing, R. Fischer-Betz, I. Haase, J. Mucke, O. Sander, J. G. Richter, T. Filla, M. Schneider, G. Chehab

**Affiliations:** 1 Department of Rheumatology, Medical Faculty of Heinrich, University Hospital Düsseldorf, Heine University, Moorenstr. 5, 40225 Düsseldorf, Germany; 2 Hiller Research Center, University Hospital Düsseldorf, Medical Faculty of Heinrich Heine University, Düsseldorf, Germany

**Keywords:** Patient-reported outcomes (PROs), Disease activity, Giant cell arteritis, Large vessel vasculitis, Takayasu arteriitis

## Abstract

**Background:**

Patient-reported outcomes (PROs) play a crucial role in assessing rheumatic diseases, offering insights into disease evaluation and treatment efficacy. This study focuses on PRO assessment in large vessel vasculitides, including Takayasu Arteritis and Giant Cell Arteritis (GCA).

**Methods:**

We retrospectively analyzed routine data from patients treated at our rheumatology clinic over a 10-year span. Patient and physician-rated global disease activity scale (G-DAS) scores, measured on a numeric rating scale (0–10 points), were collected at each visit. Clinical variables like age, sex, body mass index (BMI), disease duration, lab values, pain perception, and questionnaire responses were recorded. Linear regression and generalized additive linear regression (GAM analysis) examined associations between PROs and these factors.

**Results:**

The study included 138 patients, primarily diagnosed with GCA (94.4%). Mean follow-up was 2.5 years (0-7.7). Patient and physician G-DAS exhibited a moderate correlation (Pearson R 0.19, CI 0.14–0.24, *p* < 0.001). Higher patient G-DAS correlated with younger age (CI -3.4 - -1.5, *p* < 0.001), increased pain (CI 3.5-4, *p* < 0.001), functional limitations (HAQ, CI 0.5–0.6, *p* < 0.001), reduced physical (CI 2.3–2.7, *p* ≤ 0.001) and psychological well-being (CI 2.1–2.5, *p* < 0.001), and higher BMI (CI 1.3–2.4, *p* < 0.001). Physician G-DAS correlated with Birmingham Vasculitis Activity Score (V3.0; R 0.42, p 0.046) and were significantly linked to serum CRP elevations (β = 0.04, CI 0.0-0.08, p 0.028).

**Conclusions:**

These findings underscore the need to integrate PRO measures into vasculitis disease management strategies, enhancing the understanding of disease activity from the patient’s perspective.

**Supplementary Information:**

The online version contains supplementary material available at 10.1186/s41687-023-00681-w.

## Background

In recent years, there has been a notable paradigm shift in the field of rheumatology, acknowledging the growing significance of patient-reported outcomes (PROs) as crucial indicators for the comprehensive evaluation of rheumatic diseases. Within the context of systemic lupus erythematosus (SLE), PROs serve as a valuable source of additional information, shedding light on disease aspects that may not be adequately addressed during routine clinical assessments. By employing patient-reported questionnaires, patients are empowered. In addition, PROS are a possibility to triage follow-up appointments and enable the collection of substantial data with considerable time and cost savings [1–4].

Previous studies explored the utility of generic patient-reported outcome (PRO) instruments like the 36-item short form health survey (SF-36) in patients with large vessel vasculitis (LVV) [5–8]. However, these instruments often fail to adequately address the essential disease-specific domains that hold significant importance for patients with LVV. Recognizing the limitations of generic PROs in terms of their specificity [8, 9], the Outcome Measures in Rheumatology (OMERACT) Vasculitis Working Group acknowledged the need for a patient-reported outcome (PRO) instrument specifically designed for Giant Cell Arteritis (GCA) [10].

In 2023, the OMERACT Vasculitis Working Group successfully developed and validated the GCA-PRO questionnaire. The GCA-PRO comprehensively assesses disease-related limitations from the patient’s perspective including the subdomains ‘acute symptoms’, ‘activities of daily living’, ‘psychological’ and ‘participation’ [10]. The score of all subdomains differed between patients who reported having an active disease and those who stated that the disease was in remission. An investigation to determine whether the patients assessment correlates with the physicians assessment of disease activity as well as the analysis of associated factors is an unmet need. The evaluation of disease activity holds particular significance in terms of establishing the requirement for follow-up appointments, clinical and laboratory testing, and therapeutic decision-making.

Therefore, the objective of our study was to investigate patients’ self-assessment of disease activity on a numeric rating scale ranging from 0 to 10, correlating it with the caring physicians’ assessments, and identifying factors that may influence these assessments. The findings from our study aim to support the development of PRO measures for assessing disease activity in LVV, ultimately contributing to the optimization of disease management.

## Methods

A retrospective analysis was conducted using data collected from patients diagnosed with large vessel vasculitis, including Takayasu Arteritis (TA) and Giant Cell Arteritis (GCA), who received treatment at our rheumatology outpatient clinic over the past 10 years. At each patient visit, both patient- and physician-rated global disease activity scale (G-DAS) scores were independently determined using numeric rating scales (NRS) ranging from 0 to 10 (0 = no disease activity, 10 = maximal disease activity). The exact question that was asked to the patients at each patient visit was: *‘How do you currently assess the activity of your rheumatic disease? Please provide a value from 0 to 10 points. 0 points indicate that the disease is not active, and 10 points indicate that the disease is maximally active*.” According to this the attending physician was asked to ‘*assess the current disease activity of the patient on a scale of 0 to 10 points, where 0 points indicate that the disease is not active, and 10 points describe the maximum possible disease activity*.’ Furthermore, various clinical factors that could potentially influence these assessments were routinely recorded, such as patient’s age, sex, body mass index (BMI), disease duration, laboratory values (CRP level (mg/dl)), perception of pain (NRS 0–10), results of the Health Assessment Questionnaire (HAQ, FFBH (Funktions-Fragebogen-Hannover) derived), and patient’s subjective evaluation of physical and psychological well-being (NRS 0–10).The patient-reported data were collected on a paper questionnaire before the doctor’s visit and, following the visit, transferred into the hospital information system by two documentarians using a four-eye principle. The physician’s assessments were entered directly into the digital hospital information system during or after the patient’s visit. Thus, the physician and patient assessments of disease activity were assessed independently of each other.

The data are thus stored in the digital information system of our clinic. We exported and subsequently analyzed the visit data of all large vessel vasculitis patients from the last 10 years.

To analyze the association between patient and physician G-DAS, as well as the impact of the aforementioned variables, a linear regression analysis was performed. Random effects for patient identifier (ID) and physician ID were included in the analysis to account for repeated patient enrollments and assessments by different physicians. Additionally, a sensitivity analysis using generalized additive linear regression (GAM analysis) was conducted to capture potential nonlinear relationships between the covariates and the G-DAS. All data were analyzed using the statistical software program R (The R Foundation for Statistical Computing, Vienna, Austria, Version 4.2.1). Normal distribution was assessed visually, and descriptive statistics were reported accordingly. Normally distributed parameters are presented as mean (± standard deviation (SD)), while non-normally distributed parameters are reported as median and interquartile range (median [IQR]).

This study was conducted in accordance with ethical guidelines, and it received approval from the Heinrich-Heine-University Düsseldorf Institutional Review Board (approval number: 2021 − 1365). The study adhered to the principles outlined in the Declaration of Helsinki, ensuring the protection of participants’ rights and welfare. Consequently, no additional review or approval was deemed necessary.

## Results

We conducted a retrospective analysis involving 142 patients diagnosed with large vessel vasculitis, with the majority (n = 134, 94.4%) being diagnosed with GCA. Among the patient population, 74.6% were women, and mean age was 73.9 years (± 8.9). The average BMI was 25.9 kg/m² (± 4.2), and the mean disease duration was 8.2 years (± 5.5). Patients reported an average pain score of 3 points (± 2.8) on a NRS of 0–10 over the preceding seven days. Further details are depicted in Table 1.

**Table 1:** Characteristics of the patient cohort. Global disease activity was assessed by patients (A) and physicians (B) during 1673 clinic visits over a ten-year period. Abbreviations: SD, standard deviation; IQR, interquartile range; GCA, giant cell arteritis; TA, Takayasu arteritis, BMI, body mass index (kg/m2), HAQ, Health Assessment Questionnaire; CRP, c-reactive protein

	n (%)	Mean (SD)	Median [IQR]
**Individuals**	142 (100)		
**Visits**	1673 (100)		
**Females**	106 (74,6)		
**GCA (M31.5 + M31.6)**	134 (94.4)		
**TA (M31.4)**	8 (5.6)		
**Visits per patient**		11.78 (9.9)	9.5 [8]
**Age (years)**		73.93 (8.94)	76 [10]
**BMI (kg/m2)**		25.88 (4.16)	25.1 [6]
**Patient-assessed global disease activity score (A) (NRS 0–10)**		3. (2.5)	3 [4]
**Physician-assessed global disease activity score (B) (NRS 0–10)**		2 (1.0)	2 [2]
**Difference (A-B)**		1 (2.5)	1 [4]
**Disease duration (years)**		8.16 (5.5)	8 [7]
**Pain past 7 days (NRS 0–10)**		3 (2.8)	3 [5]
**Physical well-being (NRS 0–10)**		4 (2.0)	4 [3]
**Psychological well-being (NRS 0–10)**		3.1 (2.3)	2 [2]
**HAQ Score**		1.1 (0.6)	1 [0.9]
**CRP (mg/dl)**		0.9 (1.4)	0.4 [0.7]

**Fig. 1:**
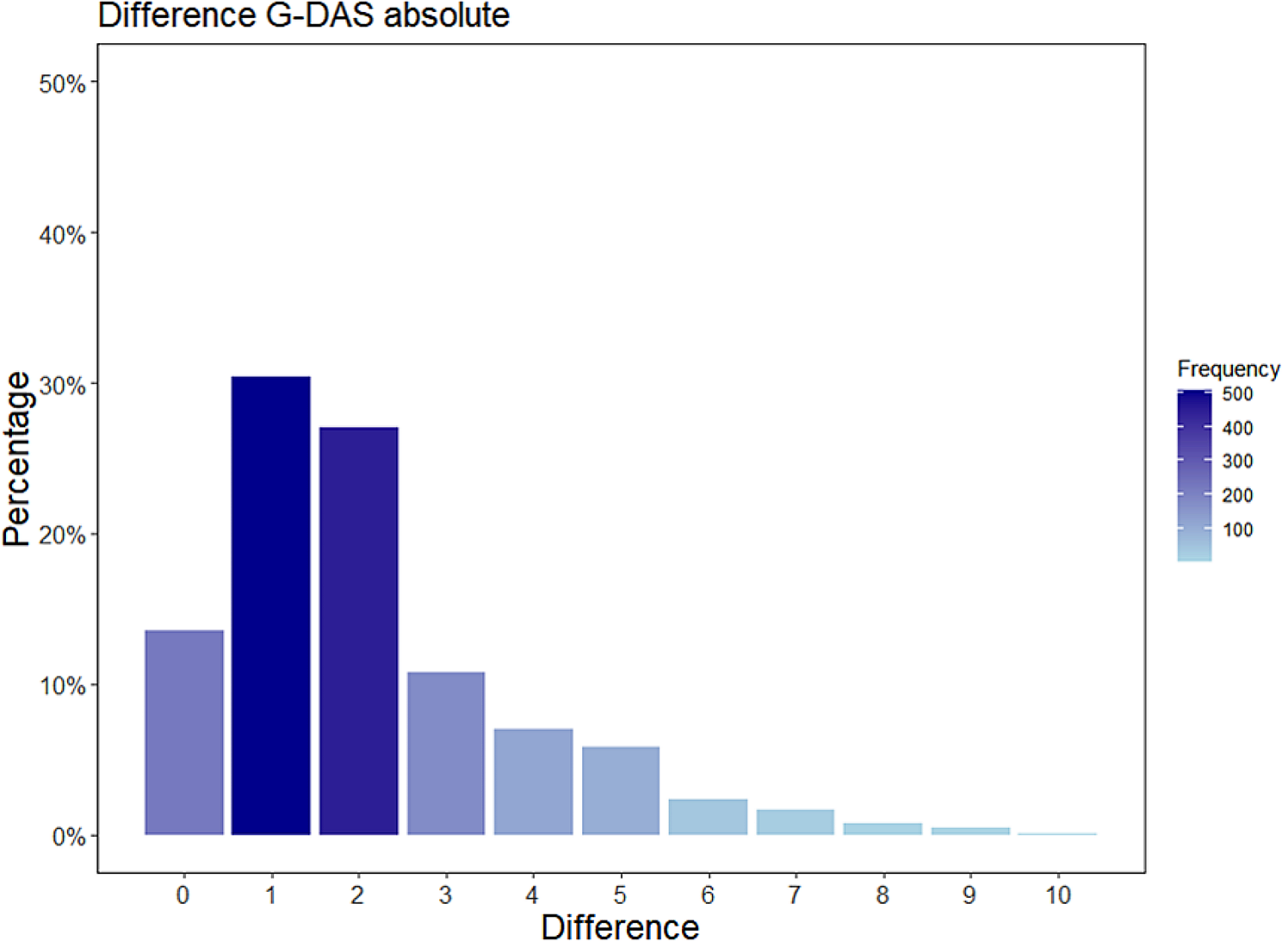
Differences in patient-assessed and physician-assessed global disease activity scores. The patient and physician-assessed scores diverged by < = 2 points in 71% (n = 1186) of the cases The number of cases contributing to the percentage values shown on the y-axis is included above each column

**Fig. 2:**
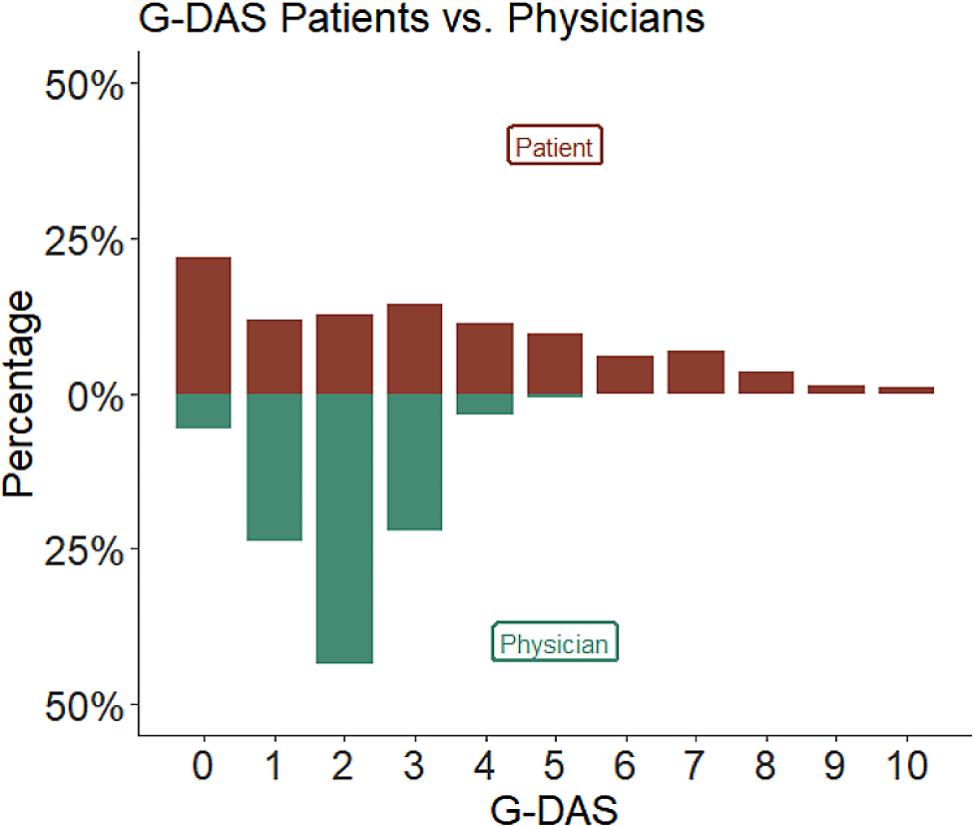
Distribution of physicians and patients global assessments of disease activity (mirror plot). Patients were more likely to assess a disease activity score of 0 points (n = 365) and greater than 5 points (n = 309)

During the outpatient visits, patients and their treating physicians independently assessed the disease activity, resulting in 1673 paired assessments. The mean follow-up time was 2.5 years, ranging from 0 to 7.7 years. On average, each patient had 11.8 outpatient visits (± 9.9) and predominantly saw the same physician during most visits (median change of attending physician: 1 [1]). When not considering any influencing factors or confounders, there was a moderate correlation (Pearson R 0.19, CI 0.14–0.24, *p* < 0.001) between patients’ and physicians’ global disease activity assessments (G-DAS). The physician’s G-DAS correlated with the physician derived BVAS Score (V3.0; *R* 0.42, p 0.046), whereas the patients’ G-DAS did not (*R* = 0.01, *p* = 0.98). The median global disease activity reported by patients was 3, compared to 2 reported by physicians. In 71.2% of cases (n = 1186), the patient and physician assessments diverged by an absolute amount of ≤ 2 points (no divergence: 13.5%, divergence of 1–2 points: 58% and divergence of more den 2 points: 29%), (see Fig. 1). Patients were more likely than physicians to report a G-DAS of 0 points (n = 365) or greater than 5 points (n = 309), as illustrated in Fig. 2. There was no meaningful difference in the patients’ and physicians’ disease activity assessments between patients diagnosed with TA and GCA. Patients and physicians G-DAS and the difference of patient’s G-DAS and physician’s G-DAS were not related to the disease duration.

Patients who reported a G-DAS ≥ 5 points were found to be younger (CI -3.4 to -1.5, *p* < 0.001). Additionally, these patients reported higher levels of pain (CI 3.5-4, *p* < 0.001), greater limitations in daily living (HAQ, CI 0.5–0.6, *p* < 0.001), poorer psychical (CI 2.1–2.5, *p* ≤ 0.001) and physical well-being (CI 2.3–2.7, *p* ≤ 0.001) compared to patients with a G-DAS of ≤ 5 points. A higher BMI was also found in the elevated G-DAS group (CI 1.3–2.4, *p* ≤ 0.001). No significant differences were observed in serum CRP levels, or disease duration between these groups.

Physicians (but not patients) reported a significantly higher G-DAS based on whether the patient experienced a flare-up during the current visit, as reported by the physician (CI 1.01–1.80, *p* ≤ 0.001). However, G-DAS from patients who never experienced a flare-up exhibited a lower correlation coefficient (R 0.14, CI 0.09–0.19, *p* ≤ 0.001) with the physician’s G-DAS compared to patients who had experienced a flare-up before (R 0.46, CI 0.33–0.56, *p* ≤ 0.001).

Linear regression analysis, with patients’ and physicians’ IDs considered as random effects, was performed to examine the impact of various factors on patient and physician assessments of global disease activity.

Among our findings, physician-documented disease activity was associated with serum CRP levels (β = 0.04, CI 0.0-0.08, p 0.028), and slightly with patients’ physical well-being (β = 0.05, CI 0.01–0.09, p 0.028).

By contrast, patient disease activity assessments were strongly associated with the degree of pain (β = 0.42, CI 0.38–0.47, *p* < 0.001) and patients’ physical and psychological well-being (β = 0.24, CI 0.18–0.31, *p* < 0.001 and β = 0.11, CI 0.06–0.16, *p* < 0.001), details are given in Table 2. A sensitivity analysis (GAM analysis) to account for nonlinear relationships between the covariates and the G-DAS of disease activity reported robust results. Relationship of variables are shown in Supplementary Figs. 1, 2.

**Table 2:** Linear regression with random effects of patient and physician assessments of global Disease activity. G-DAS, global disease activity scale; BMI, body mass index (kg/m2); CRP, C-reactive protein; HAQ, health assessment questionnaire; NRS, numeric rating scale; CI (95%) 95% Confidence interval

	G-DAS patients			G-DAS physicians		
	Estimates	CI (95%)	*p*	Estimates	CI (95%)	*p*
**(Intercept)**	-0.31	-2.51–1.89	0.785	1.78	0.63–2.94	0.003
**Age**	0.00	-0.02–0.02	0.928	-0.01	-0.02–0.00	0.263
**BMI**	0.01	-0.03–0.06	0.577	0.00	-0.02–0.02	0.918
**Sex [female]**	0.09	-0.42–0.59	0.734	-0.00	-0.26–0.25	0.979
**G-DAS physicians**	0.04	-0.06–0.14	0.412			
**G-DAS patients**				0.01	-0.02–0.05	0.407
**HAQ-Score**	0.27	0.00–0.53	0.048	0.01	-0.13–0.16	0.870
**Physical well-being (NRS 0–10)**	0.24	0.18–0.31	< 0.001	0.05	0.01–0.09	0.010
**Psychological well-being (NRS 0–10)**	0.11	0.06–0.16	< 0.001	0.01	-0.02–0.04	0.377
**Pain last 7 days (NRS 0–10)**	0.42	0.38–0.47	< 0.001	0.02	-0.01–0.05	0.143
**Disease duration**	0.02	-0.02–0.06	0.335	0.01	-0.01–0.04	0.186
**CRP (mg/dl)**	-0.11	-0.18 – -0.05	0.010	0.04	0.00–0.08	0.028

## Discussion

Patient-reported outcomes for assessing disease-related limitations and disease activity from the patient’s perspective have gained importance in evaluating various rheumatic diseases, including LVV [2, 10–13]. In LVV generic and disease specific PROMs exist. While generic PROMs offer the advantage of facilitating comparisons across diverse disease groups, they may lack sensitivity to disease-specific questonnaries. So far the generic PROs used in LVV are the Short-Form-36 (SF-36) [14] and the Euroqol (EQ-5D-5 L) [15]. As the SF-36 demonstrates poor correlation with ocular involvement in GCA, the effectiveness as a standalone outcome measure in clinical trials is compromised [8, 10].

Thus, in 2015 the Large Vessel Vasculitis Working Group emphasized the necessity for a disease-specific PROM dedicated to GCA [16], that was published in 2023 (GCA-PRO) [10]. So far, the GCA-PRO is the only disease-specific PRO (Patient-Reported Outcome) in LVV. It provides a comprehensive evaluation of the impact of GCA on the domains ‘acute symptoms’, ‘activities of daily living’, ‘psychological’ and ‘participation’. The score of all four subdomains differed between patients who reported having an active disease and those who stated that the disease was in remission. So far, there was no existing comparison of the assessment of disease activity between the physician and the patient, as well as the influencing factors for each assessment, for LVV. However, the assessment of the current disease activity from a patient’s perspective is still an unmet need and can be a tool to triage follow-up appointments and monitor treatment response.

Therefore, we aimed to investigate the global disease activity assessment using a numeric rating scale assessed by both, patients diagnosed with LVV and their physicians. Our study contributes to understand ways to assess patients disease activity considering influencing factors in order to integrate these findings into future research and disease management.

We confirmed that the Birmingham Vasculitis Activity Score (BVAS), a validated tool to assess disease activity by physicians, correlates with their global disease activity assessment, as described before [17].

When comparing the assessments of disease activity between patients and physicians, we observed that the majority of patients (70,9%) and their physicians had similar evaluations, with a median difference of ≤ 2 points. However, in just 13,5% of the visits physicians and patients reported the exact same G-DAS. Moreover, patients tended to report higher disease activity levels compared to physicians, a finding that is consistent with previous studies on other rheumatic diseases, [18, 19]. The correlation between patient and physician assessments was stronger when disease activity was relatively low. This trend is in line with findings from studies on SLE and Rheumatoid Arthritis, indicating that the agreement between patient and physician assessments diminishes at higher disease activity levels [3, 19, 20].

Our analysis revealed that physicians’ assessments of disease activity were significantly associated with serum CRP levels. On the other hand, patient assessments were influenced by pain intensity and overall physical and psychological well-being. Particularly when the G-DAS exceeded 5 points, these subjective factors had a strong impact on patients’ assessments of disease activity. Comparable observations have already been described for other rheumatic diseases: In SLE, patients are more likely to rate their disease activity on the basis of their psychological and physical well-being [21, 22] and measure the treatment success on the basis of practical effects on their functionality in daily life [23].

Thus, we recommend that healthcare providers address pain and physical well-being directly during physician-patient conversations to better understand their influence on the patient’s perception of disease activity. By addressing these aspects, appropriate strategies such as optimized analgesic therapy and physiotherapy should be discussed to improve patients’ overall quality of life.

It is important to acknowledge the limitations of our study. Due to its retrospective nature and the data collection spanning a 10-year period, we were unable to compare global disease activity and BVAS with the specific domains outlined in the GCA-PRO. Additionally, our study cohort did not allow us to assess the prediction of flares based on patients’ global disease activity assessments, as we only had limited data (4 patients) about the G-DAS within 0–3 month prior a flare. Further prospective studies are needed to address these gaps and provide more comprehensive insights. This includes a prospective validation of the numeric physicians and patients G-DAS, the GCA-PRO and the BVAS conducting a longitudinal multicenter data analysis including the assessment of the described factors that influenced the patients and physicians G-DAS.

## Conclusion

In conclusion, our study demonstrated that patients and physicians generally agreed on the assessment of disease activity in LVV, although patients tended to report higher levels of disease activity. Different factors influenced the assessments, with physicians’ evaluations being associated with objective measures like CRP levels, while patients’ assessments were influenced by subjective factors such as pain and the general and psychological well-being. Our findings emphasize the importance of developing and evaluating PROs to accurately assess disease activity in patients diagnosed with LVV and offer insights for optimizing disease management.

### Electronic supplementary material

Below is the link to the electronic supplementary material.

Supplementary Material 1

Supplementary Material 2

## Data Availability

All data relevant to the study are included in the article. Data are available on reasonable request. The data will be shared if there is a reasonable request for it.

## Declarations

### Patient and public involvement

The retrospective analysis was conducted using data collected from patients diagnosed with large vessel vasculitis, including Takayasu Arteritis (TA) and Giant Cell Arteritis (GCA), who received treatment at our rheumatology outpatient clinic over the past 10 years.

## Abbreviations

PROs Patient: Reported outcomes
TA: Takayasu Arteritis
GCA: Giant Cell Arteritis
G-DAS: Global disease activity scale
NRS: Numeric rating scale
BMI: Body mass index
GAM: Generalized additive linear regression
HAQ: Health assessment questionnaire
CRP: c-reactive protein
SLE: Systemic lupus erythematosus
SF-36: 36-item short form health survey
LVV: Large vessel vasculitis
OMERACT: Outcome Measures in Rheumatology
ID: Identifier
SD: Standard deviation
IQR: Interquartile range
BVAS: Birmingham vasculitis activity score
CI: confidence interval
GCA-PRO: Giant Cell Arteritis-patient reported outcome
FFBH: Funktions-Fragebogen-Hannover
