# Supplementary figures and images for: Patient-reported outcomes in large vessel vasculitis: insights from a retrospective analysis of disease activity and associated factors
Source: J Patient Rep Outcomes. 2024 Jan 8;8:4. doi: 10.1186/s41687-023-00681-w (PMC10825095; doi:10.1186/s41687-023-00681-w)

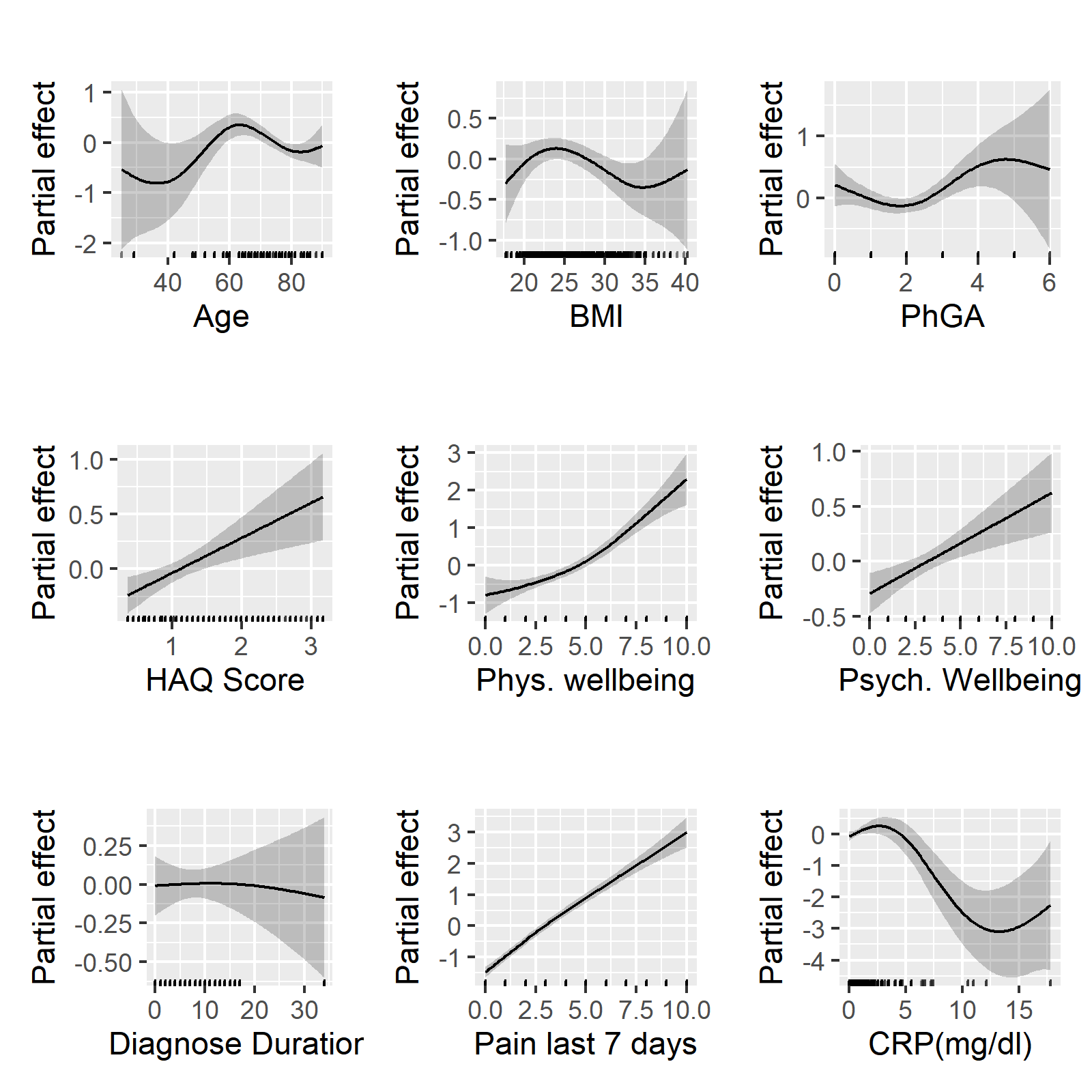

Supplement: Supplementary file 1 — Supplementary Material 1 [file 41687_2023_681_MOESM1_ESM.tif]

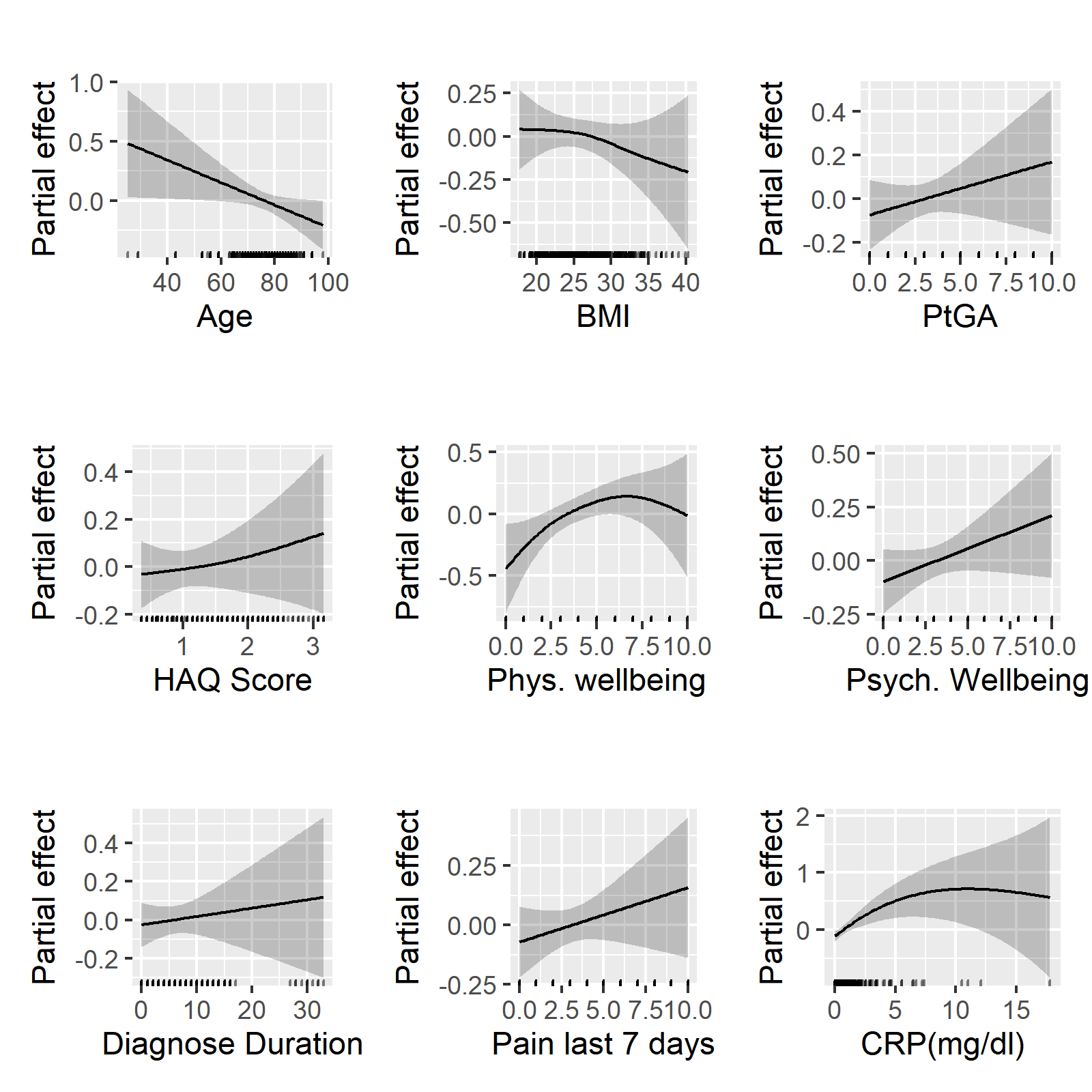

Supplement: Supplementary file 2 — Supplementary Material 2 [file 41687_2023_681_MOESM2_ESM.tif]
